# Distinct mobilization of leukocytes and hematopoietic stem cells by CXCR4 peptide antagonist LY2510924 and monoclonal antibody LY2624587

**DOI:** 10.18632/oncotarget.21816

**Published:** 2017-10-10

**Authors:** Sheng-Bin Peng, Robert D. Van Horn, Tinggui Yin, Robin M. Brown, William C. Roell, Victor H. Obungu, Charles Ruegg, Victor J. Wroblewski, Eyas Raddad, John R. Stille

**Affiliations:** ^1^ Lilly Research Laboratories, Eli Lilly and Company, Indianapolis, Indiana 46285, USA; ^2^ The Chorus Group, Eli Lilly and Company, Indianapolis, Indiana 46285, USA

**Keywords:** CXCR4, peptide antagonist, LY2510924, monoclonal antibody, LY2624587

## Abstract

Stromal cell-derived factor-1 (SDF-1) and its receptor CXCR4 play a critical role in mobilization and redistribution of immune cells and hematopoietic stem cells (HSCs). We evaluated effects of two CXCR4-targeting agents, peptide antagonist LY2510924 and monoclonal antibody LY2624587, on mobilizing HSCs and white blood cells (WBCs) in humans, monkeys, and mice. Biochemical analysis showed LY2510924 peptide blocked SDF-1/CXCR4 binding in all three species; LY2624587 antibody blocked binding in human and monkey, with minimal activity in mouse. Cellular analysis showed LY2624587 antibody, but not LY2510924 peptide, down-regulated cell surface CXCR4 and induced hematological tumor cell death; both agents have been shown to inhibit SDF-1/CXCR4 interaction and downstream signaling. In animal models, LY2510924 peptide induced robust, prolonged, dose- and time-dependent WBC and HSC increases in mice and monkeys, whereas LY2624587 antibody induced only moderate, transient increases in monkeys. In clinical trials, similar pharmacodynamic effects were observed in patients with advanced cancer: LY2510924 peptide induced sustained WBC and HSC increases, while LY2624587 antibody induced only minimal, transient WBC changes. These distinct pharmacodynamic effects in two different classes of CXCR4 inhibitors are clinically important and should be carefully considered when designing combination studies with immune checkpoint inhibitors or other agents for cancer therapy.

## INTRODUCTION

The chemokine and chemokine receptor pair of stromal cell-derived factor-1 (SDF-1; also called CXCL12) and chemokine (C-X-C) motif receptor 4 (CXCR4) plays a critical role in leukocyte trafficking, immune response, and tumorigenesis [1–3]. CXCR4 is often expressed in tumor cells with its overexpression associated with poor prognosis in many cancer types, and SDF-1 is highly expressed in tumor microenvironments [4–6]. Interaction of SDF-1/CXCR4 plays an important role in tumor cell proliferation, survival, invasion, metastasis, and immune suppression [4–7]. Preclinical and emerging clinical data suggest CXCR4 is an attractive target for antitumor drug development [5]. Agents that target CXCR4, including small molecule inhibitors, monoclonal antibodies (mAbs), and peptide antagonists, have been shown to disrupt SDF-1/CXCR4 binding and downstream signaling with therapeutic applications in cancer and immunopathology. A variety of such agents are currently being evaluated in clinical trials [7–14].

In addition to tumor microenvironments, SDF-1 is constitutively expressed at high levels in bone marrow and is an efficient chemotactic factor for immune cells including T-cells, B-cells, and monocytes [1, 2, 5, 15–17]. Preclinical and clinical studies of agents that target CXCR4, including small molecule inhibitors, peptide antagonists, peptide agonists, and modified SDF-1, have demonstrated mobilization of immune cells and hematopoietic stem cells (HSCs) into peripheral blood by altering cell homing, retention, and release from the bone marrow compartment [18–24]. Several CXCR4-targeting agents, small molecule inhibitor AMD3100 (also called plerixafor) and peptide antagonist BKT140, mobilize both white blood cells (WBCs) including neutrophils and HSCs [12, 20, 24]. Clinically, AMD3100 is used to mobilize HSCs in patients with multiple myeloma and non-Hodgkin lymphoma [13, 25, 26].

We developed LY2624587, a fully humanized CXCR4 mAb that is among the first-in-class anti-CXCR4 mAbs developed, for the treatment of patients with cancer [10]. LY2624587 potently blocks SDF-1 binding to CXCR4, inhibits SDF-1/CXCR4 mediated cell signaling including ERK and AKT activation, induces cell surface receptor internalization, and induces dose-dependent cell death *in vitro* and *in vivo* in human hematologic cancer cells. LY2624587 has no independent agonist activity [10]. A role for LY2624587 antibody in mobilizing HSCs and WBCs has not been previously reported. We also developed LY2510924, a novel cyclic peptide antagonist that potently and selectively blocks SDF-1/CXCR4 interaction and downstream signaling [8]. In preclinical models of solid tumors and acute myeloid leukemia, LY2510924 peptide effectively disrupted SDF-1/CXCR4 signaling to induce antitumor effects as a monotherapy and was enhanced in combination with chemotherapy [8, 27]. In a phase I clinical trial in patients with advanced cancer, LY2510924 peptide mobilized CD34^+^ HSCs and neutrophils with favorable pharmacokinetic and safety profiles [9].

Immune checkpoint therapies target regulatory pathways in T-cells to enhance antitumor immune responses, and have led to significant clinical advances for treatment of cancer [28]. However, these therapies have elicited durable clinical responses and long-term remissions in only a fraction of patients, suggesting that combination regimens may be needed [28, 29]. Due to the critical role of SDF-1/CXCR4 interaction in immune cell retention and mobilization, CXCR4 inhibition may lead to T-cell infiltration and redistribution in tumor microenvironments. Indeed, mice with pancreatic cancer had rapid T-cell accumulation near tumors induced by small molecule inhibitor AMD3100, which was synergistic with an anti–PD-L1 mAb to eliminate tumor cells [7]. In hepatic carcinoma models, inhibition of CXCR4 by AMD3100 augmented anti–PD-1 combination therapy efficacy via concomitant targeting of hypoxic and immunosuppressive microenvironments [30]. Blockade of SDF-1/CXCR4 in ovarian cancer using an oncolytic vaccinia virus vector expressing a CXCR4 antagonist inhibited tumor growth by reduction of immunosuppression and targeting of tumor-initiating cells [31]. AMD3100 treatment in ovarian cancer models increased tumor apoptosis with selective reduction of intra-tumor regulatory T-cells and increased T-cell mediated antitumor immune responses [32].

There are currently several CXCR4-targeting agents, including peptide antagonists and mAbs, being evaluated in combination with checkpoint blockade for cancer immunotherapy. In multiple *in vitro* and *in vivo* studies, we evaluated two agents, LY2510924 peptide and LY2624587 antibody, for their capabilities to mobilize WBCs and HSCs in mice, monkeys, and human clinical trial patients with advanced cancer. Both agents block SDF-1 binding to CXCR4 and downstream cell signaling, but here we report findings from preclinical and clinical studies showing distinct cellular functions and pharmacodynamic responses for LY2510924 peptide and LY2624587 antibody in the mobilization of peripheral WBCs and HSCs. These important pharmacodynamic differences in the magnitude and durability of immune cell mobilization may be useful as key inputs into the design of future clinical trials investigating combined immunotherapy to treat patients with advanced cancer and hematopoietic malignancies.

## RESULTS

### Inhibitory functions of LY2510924 peptide and LY2624587 antibody *in vitro*

**Figure 1:**
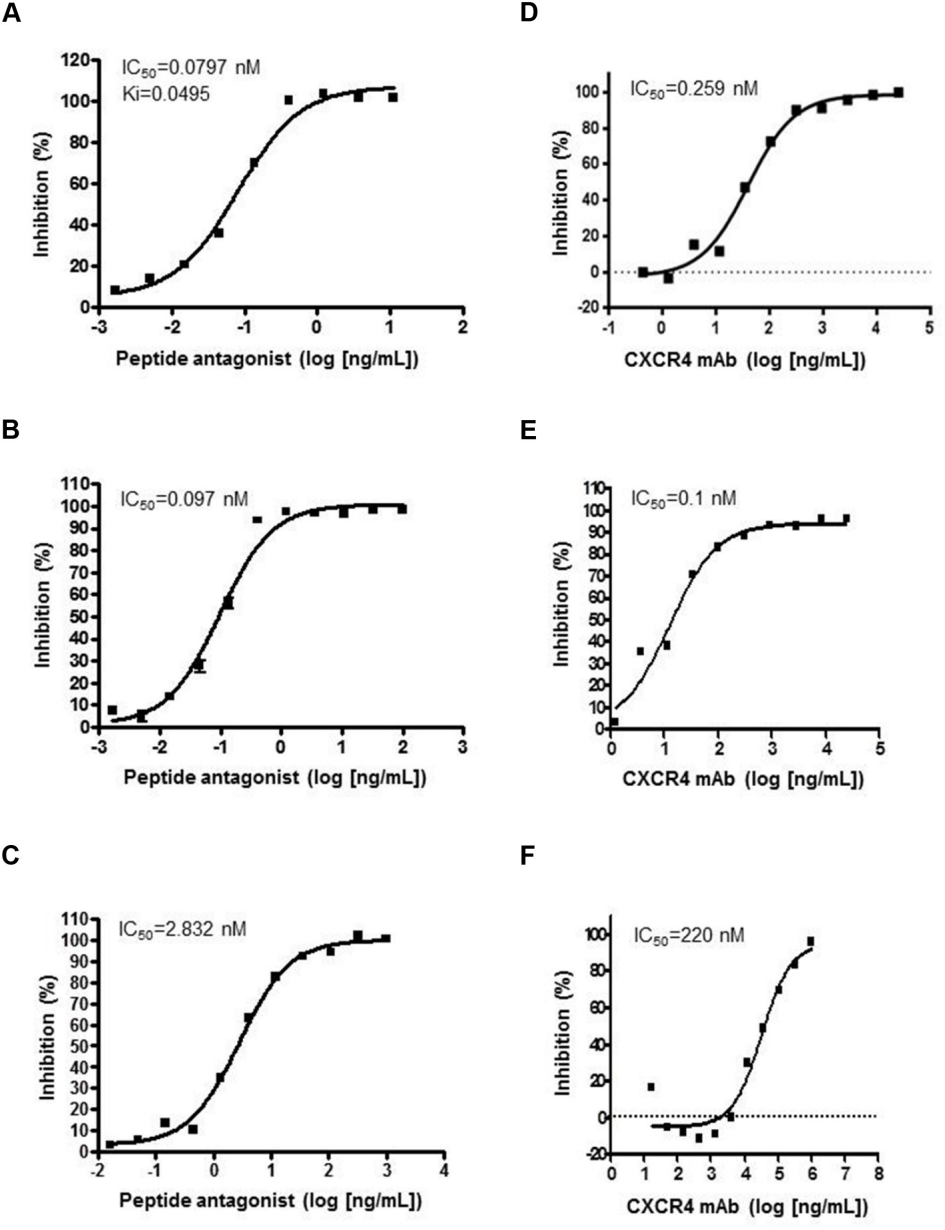
LY2510924 peptide and LY2624587 antibody inhibition of SDF-1/CXCR4 binding in human, monkey, and mouse cells LY2510924 (peptide antagonist) inhibits binding in **(A)** human, **(B)** monkey, and **(C)** mouse cells. LY2624587 (CXCR4 mAb) inhibits binding in **(D)** human, **(E)** monkey, and **(F)** mouse cells. Human cells = leukemia CCRF-CEM cells with high-level expression of human CXCR4, monkey cells = MDA-MB-435 cells stably transfected with monkey CXCR4, and mouse cells = 2PK-3 lymphoma cells with high-level expression of mouse CXCR4. Ki = inhibitor constant.

Preclinical characterization of both agents was previously described [8, 10]. Interaction of SDF-1/CXCR4 was assayed in human leukemia CCRF-CEM cells expressing high levels of human CXCR4, MDA-MB-435 cells stably transfected with monkey CXCR4, and mouse 2PK-3 lymphoma cells expressing high levels of mouse CXCR4. LY2510924 peptide inhibited SDF-1 binding in human (Figure 1A), monkey (Figure 1B), and mouse (Figure 1C) cells in a concentration-dependent manner with half-maximal inhibitory concentration (IC_50_) values of 0.08, 0.097, and 2.83 nM, respectively. LY2510924 peptide inhibition in mouse cells was less robust than in human and monkey cells with 35- and 29-fold IC_50_ differences, respectively. LY2624587 antibody inhibited SDF-1 binding in human (Figure 1D) and monkey (Figure 1E) cells with IC_50_ values of 0.26 and 0.1 nM, respectively. However, LY2624587 antibody had weaker inhibition in mouse cells with an IC_50_ of 220 nM (Figure 1F), 846- and 2200-fold differences versus human and monkey cells, respectively (Supplementary Figure 1). LY2624587 antibody does not cross-react sufficiently with mouse CXCR4 to functionally inhibit SDF-1 binding, and was not evaluated in mouse *in vivo* models.

### Cellular activities of LY2510924 peptide and LY2624587 antibody *in vitro*

**Figure 2:**
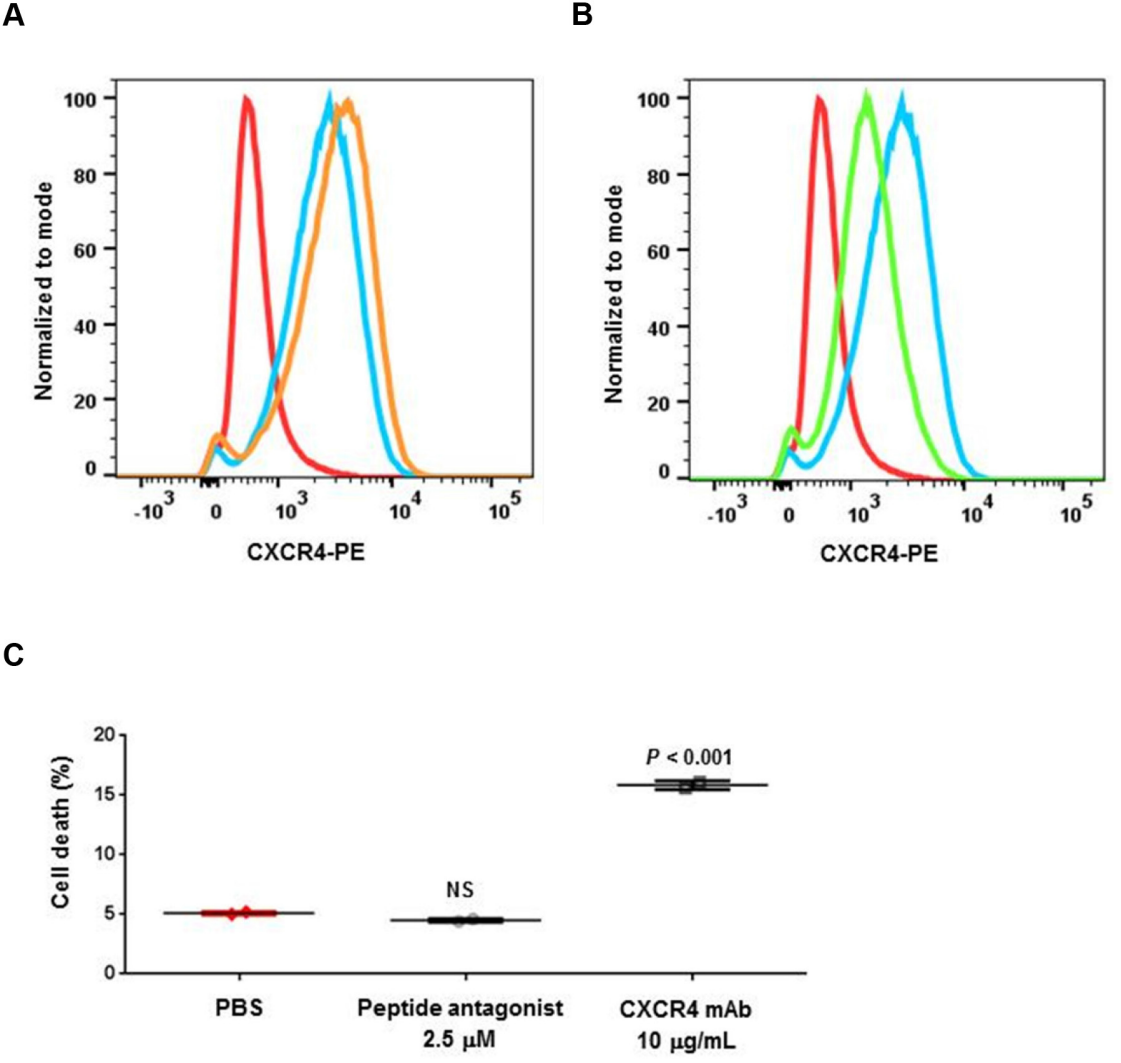
Cellular activities of LY2510924 peptide and LY2624587 antibody Flow cytometry analysis of cell surface CXCR4 protein expression (PE) following exposure to **(A)** LY2510924 (peptide antagonist) and **(B)** LY2624587 (CXCR4 mAb). Isotype control Ab = red, untreated cells = blue, treated cells = orange (A) or green (B). **(C)** Human B cell lymphoma Raji cell death 48 hours following exposure to LY2510924 (peptide antagonist) or LY2624587 (CXCR4 mAb) versus PBS control. NS = not significant.

We previously tested both agents with a panel of *in vitro* assays for ligand binding, GTP binding, cell migration, and cell signaling inhibition in tumor cells; in most of these assays, both agents showed similar biochemical and *in vitro* cellular activities [8, 10]. In the present study, we identified differences between these agents in cellular functions. Flow cytometry analysis showed LY2624587 antibody induced receptor mediated internalization and downregulation of cell surface CXCR4 in human B cell lymphoma Raji cells (Figure 2A). This is consistent with our previous results in human Namalwa non-Hodgkin lymphoma cells [10]. However, LY2510924 peptide did not induce cell surface CXCR4 downregulation; in contrast, it induced minimal upregulation of CXCR4 (Figure 2B). Additionally, these two agents showed differences in induction of cell death in Raji cells. LY2624587 antibody induced statistically significant cell death compared with PBS control (*P* < 0.001), whereas LY2510924 peptide did not (Figure 2C). LY2624587 antibody induced apoptosis in leukemia and lymphoma cells is consistent with previous observations [10].

### Mobilization of WBCs and HSCs in mice

**Figure 3:**
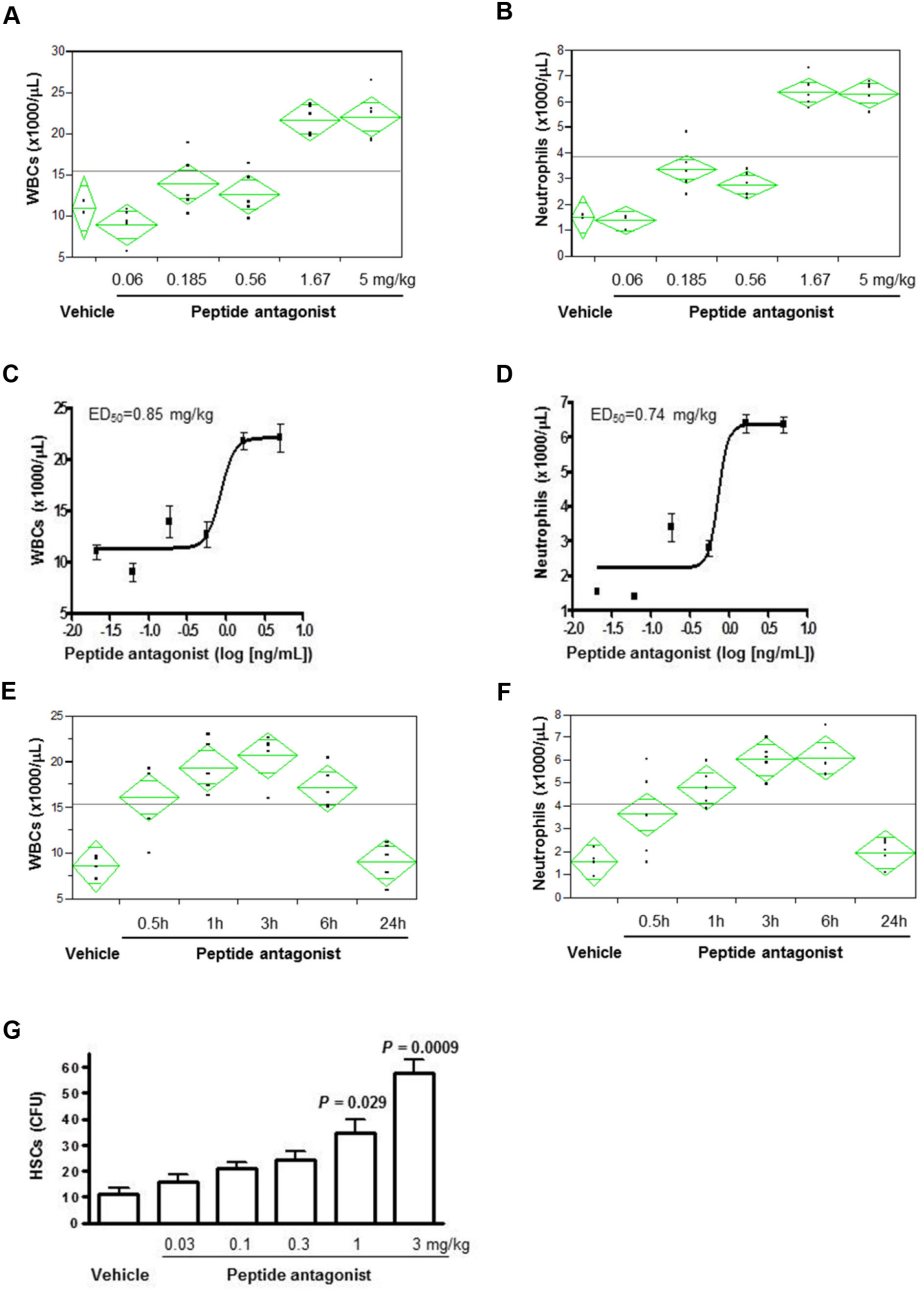
LY2510924 peptide induced mobilization of WBCs, neutrophils, and HSCs in mice Analysis of LY2510924 (peptide antagonist) induced, dose-dependent changes vs. vehicle at 3 hours in **(A)** WBCs and **(B)** neutrophils. ED_50_ of LY2510924 (peptide antagonist) induced changes in **(C)** total WBCs and **(D)** neutrophils. Analysis of LY2510924 (peptide antagonist) induced, time-dependent changes at 5 mg/kg vs. vehicle (3 hours) in **(E)** total WBCs and **(F)** neutrophils. **(G)** HSC mobilization induced by LY2510924 (peptide antagonist) acetate salt form (LSN2534820) measured by CFU.

LY2510924 peptide elicited a dose-dependent, 2- to 4-fold increase in peripheral total WBC and neutrophil counts (Figure 3A, 3B), with median effective dose (ED_50_) values of 0.85 mg/kg and 0.74 mg/kg, respectively (Figure 3C, 3D), up to the highest dose of 5 mg/kg and measured at 3 hours post-dose. A single dose of LY2510924 peptide at 5 mg/kg induced significant time-dependent increases in total WBCs and neutrophils as early as 30 minutes post-dose, with maximum effects achieved at 3-6 hours post-dose in mice (Figure 3E, 3F). WBC and neutrophil counts returned to near baseline levels by 24 hours post-dose. LY2510924 peptide administered in its acetate salt form induced robust HSC mobilization in mice in a dose-dependent manner (Figure 3G) at 3 hours post-dose, which was statistically significant versus vehicle control at doses of 1 mg/kg (*P* = 0.029) and 3 mg/kg (*P* = 0.0009), and did not appear to saturate at 3 mg/kg.

### Mobilization of WBCs and HSCs in monkeys

**Figure 4:**
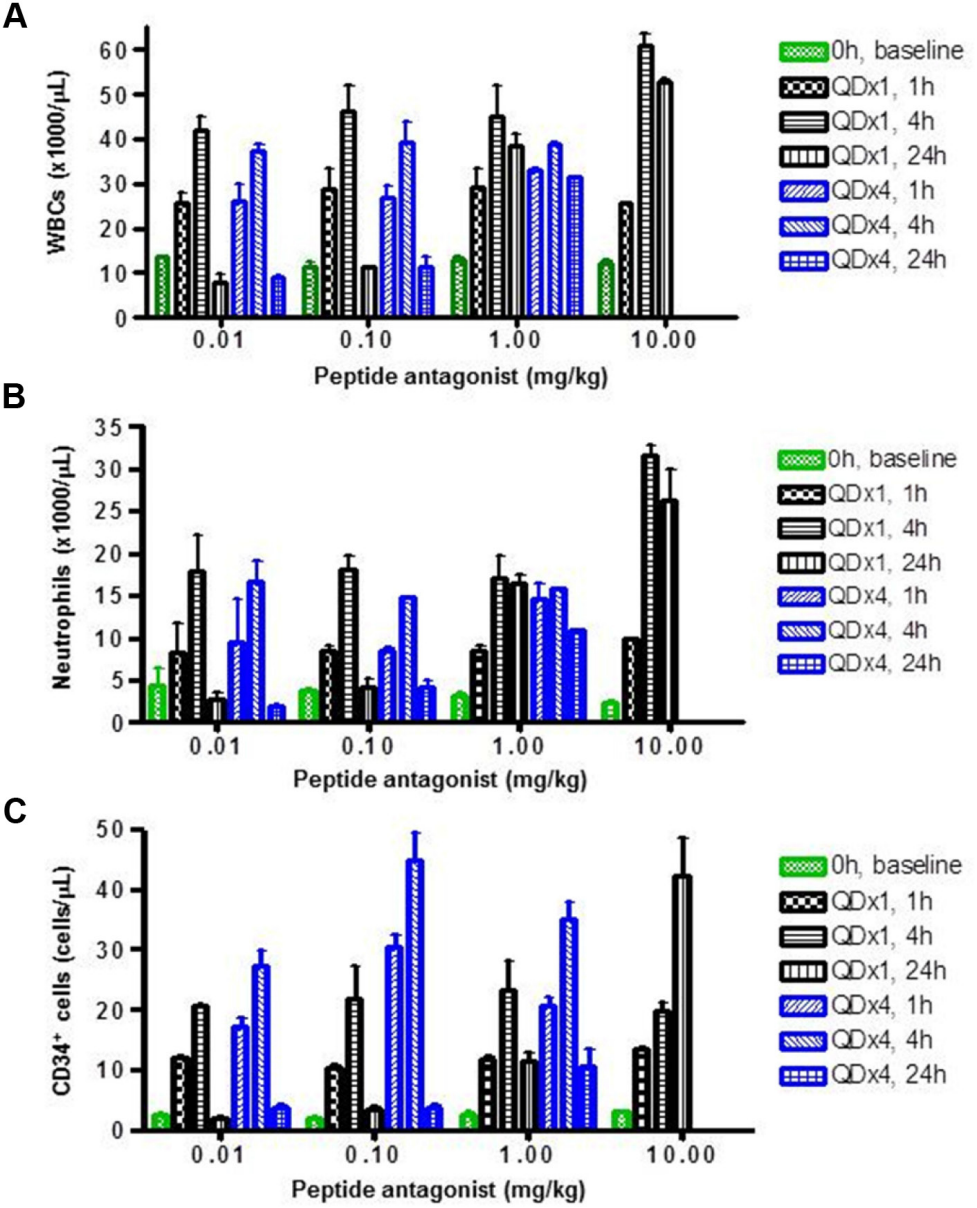
LY2510924 peptide induced mobilization of WBCs, neutrophils, and HSCs in monkeys LY2510924 (peptide antagonist) induced dose- and time-dependent changes in **(A)** total WBCs, **(B)** neutrophils, and **(C)** CD34^+^ HSCs. QDx1 = single dose; QDx4 = once daily for 4 days.

LY2510924 peptide induced dose- and time-dependent increases of WBCs and neutrophils in monkeys (Figure 4A, 4B). Significant increases in WBCs and neutrophils were observed at the lowest dose, 0.01 mg/kg; maximum increases in WBCs and neutrophils were observed at 4 hours at lower doses (0.01 and 0.1 mg/kg). In these two lower dose groups, WBC and neutrophil counts returned to near baseline levels 24 hours post-dose. However, at higher doses (1 and 10 mg/kg), these pharmacodynamic effects lasted 24 hours. Importantly, findings at day 1 (single-dose treatment) and day 4 (once-daily dosing for 4 days) were similar, and the repeated-dose schedule did not appear to alter the effects (Figure 4A, 4B). Similar to WBCs and neutrophils, LY2510924 peptide administered in its acetate salt form induced a dose- and time-dependent increase of CD34^+^ HSCs in monkeys (Figure 4C). Increases of 6- to 20-fold over baseline were observed for all dose groups at 1 and 4 hours post-dose; at higher doses (1 and 10 mg/kg) the increases lasted 24 hours. Unlike observations for WBCs and neutrophils, the repeated-dose schedule (once daily for 4 days) appeared to increase the magnitude of CD34^+^ HSC mobilization.

**Figure 5:**
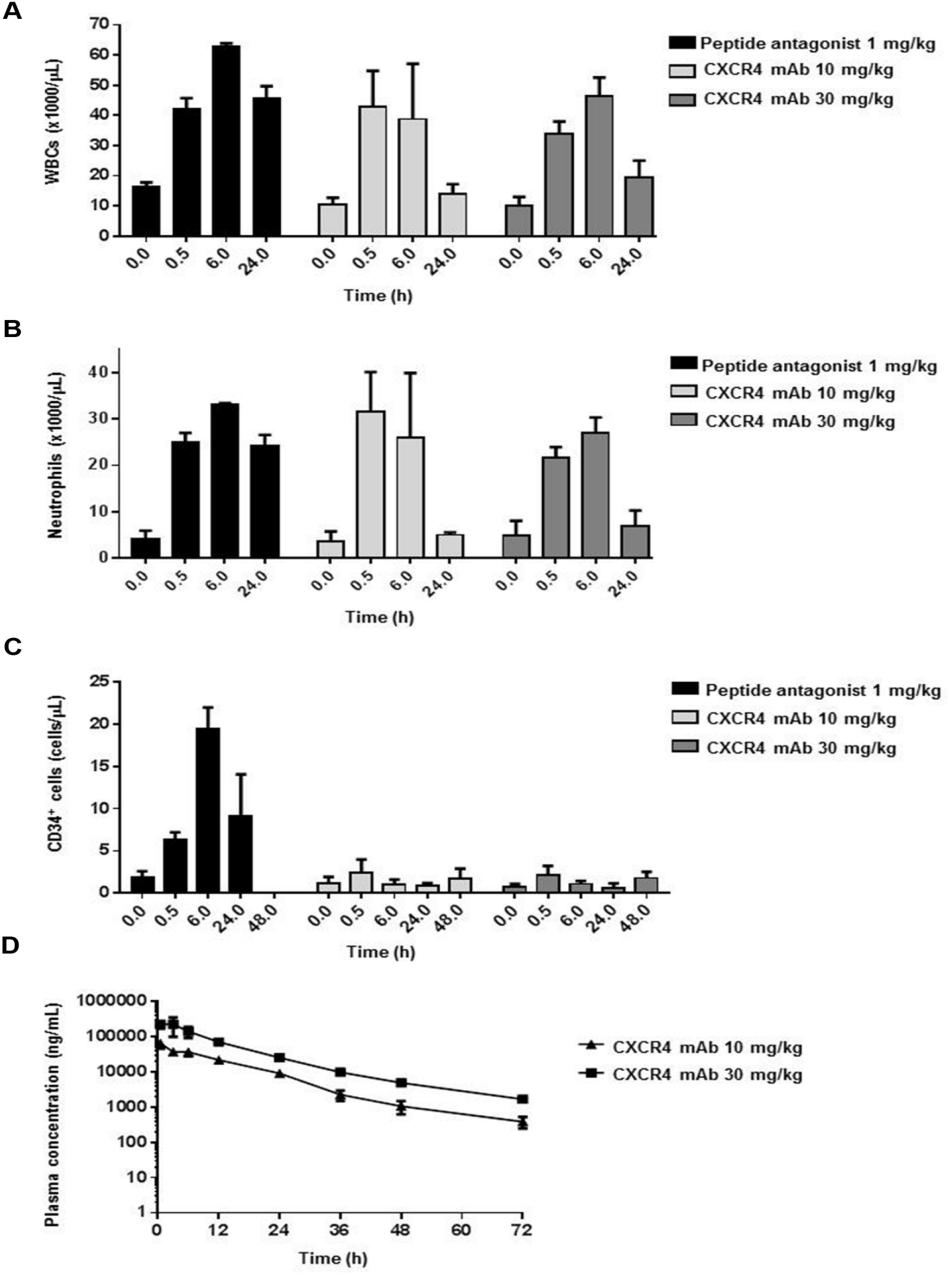
LY2624587 antibody induced mobilization of WBCs, neutrophils, and HSCs in monkeys LY2624587 (CXCR4 mAb) induced changes in **(A)** total WBCs, **(B)** neutrophils, and **(C)** CD34^+^ HSCs, vs. positive control LY2510924 (peptide antagonist) acetate salt form (LSN2534820). **(D)** LY2624587 (CXCR4 mAb) plasma concentrations after 10- or 30-mg/kg single doses.

LY2624587 antibody induced a significant increase in peripheral WBCs and neutrophils in monkeys in both 10- and 30-mg dose groups (Figure 5A, 5B). The increase was transient, observed only at 0.5 and 6 hours, and WBC and neutrophil counts returned to near baseline levels 24 hours post-dose. Results were similar for LY2624587 antibody at doses of 10 and 30 mg/kg, suggesting that 10 mg/kg may have saturated the pharmacodynamic effect in monkeys. In contrast, a single dose (1 mg/kg) of LY2510924 peptide administered in its acetate salt form as a positive control led to a prolonged, sustained increase in WBCs and neutrophils up to 24 hours. LY2624587 antibody doses of 10 and 30 mg/kg induced near-absent or minimal change in CD34^+^ HSCs at any time point, while the LY2510924 peptide induced a robust increase lasting up to 24 hours (Figure 5C).

To verify these pharmacodynamic results, we conducted a plasma exposure analysis to ensure we achieved sufficient exposure to LY2624587 antibody, and demonstrated that doses of 10 and 30 mg/kg achieved very high plasma exposure (Figure 5D). The 10 mg/kg dose had mean plasma exposures of 9126 and 1063 ng/mL (60.8 nM and 7.1 nM) at 24 and 48 hours, 608- and 71-fold greater than the IC_50_ (0.1 nM), respectively. The 30 mg/kg dose group had plasma exposures of 25426 and 4954 ng/mL (169.5 and 33 nM) at 24 and 48 hours, 1695- and 330-fold greater than the IC_50_, respectively. These analyses revealed a key pharmacodynamic difference between LY2510924 peptide and LY2624587 antibody in monkeys. LY2510924 peptide led to robust and prolonged mobilization of WBCs and HSCs *in vivo*, compared with the transient increase in WBCs and lack of effect on CD34^+^ HSCs induced by LY2624587 antibody; this difference was observed despite *in vitro* evidence that both agents inhibit monkey SDF-1/CXCR4 with equal potency and despite pharmacokinetic evidence of adequate *in vivo* exposure to LY2624587 antibody.

### Mobilization of WBCs and HSCs in human patients with advanced cancer

**Figure 6:**
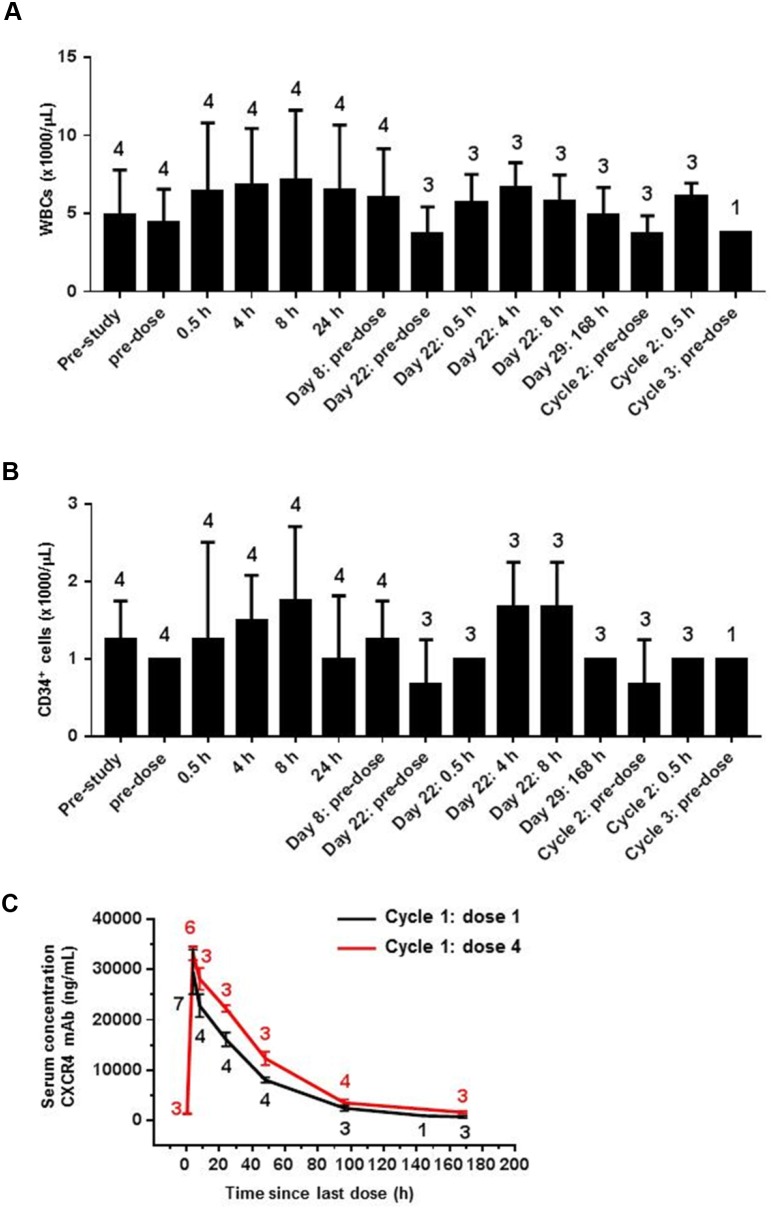
LY2624587 antibody induced mobilization of WBCs and HSCs in human clinical trial patients with advanced cancer LY2624587 (CXCR4 mAb) induced changes in **(A)** total WBCs and **(B)** CD34^+^ HSCs at study baseline and cycles 1 to 3 of 900-mg once-weekly dosing. **(C)** LY2624587 (CXCR4 mAb) serum concentrations after 900-mg once-weekly dose. Values above or below data points indicate number of patients in standard error computation.

Both LY2510924 peptide and LY2624587 antibody have advanced to clinical studies to treat advanced cancer. In phase I dose-escalation and dose-confirmation studies of both agents in patient with advanced cancer, circulating WBCs, neutrophils, and CD34^+^ HSCs were evaluated as clinical biomarkers. As previously reported, LY2510924 peptide administered over a range of 1- to 30-mg/day dosing had a favorable pharmacokinetic profile and induced dose-dependent increases in neutrophils and CD34^+^ HSCs in patients with advanced cancer [9]. Consistent with preclinical data in mice and monkeys, LY2510924 peptide treated patients had 2- to 3-fold increases in neutrophils and up to 18-fold increases in CD34^+^ HSCs with effects noted on cycle 1 day 2 and maintained through day 28 (end of treatment) [9]. LY2624587 antibody induced changes in WBC and CD34^+^ HSC counts were assessed at the 900-mg once-weekly dose level, pre- and post-dose, throughout the first cycle (dosing on days 1, 8, 15, and 22) and after end of the first treatment cycle (day 29). Further time points included pre-dose and immediately post first dose for cycle 2, and pre-dose at the start of cycle 3. In contrast to effects of LY2510924 peptide, 900 mg once-weekly LY2624587 antibody led to only transient,<1.5-fold increases in WBCs and no discernable change in CD34^+^ HSCs throughout the treatment period when compared with pre-dose or pre-study values (Figure 6A, 6B).

Pharmacokinetic analysis of cycle 1 serum concentrations showed that the 900-mg LY2624587 antibody dose and regimen achieved sufficient exposure in cancer patients; at 24 and 48 hours post first dose, plasma exposures were 16094 and 8068 ng/mL (110 and 55 nM), 425- and 212-fold higher than the IC_50_ (0.259 nM), respectively. At 24 and 48 hours post fourth dose, plasma exposures were 22248 and 12333 ng/mL (152 and 84 nM), 587- and 324-fold higher than the IC_50_, respectively (Figure 6C). Analyses conducted pre-dose and 0.5 hours post first and fourth doses showed that LY2624587 antibody at the 900-mg dose level corresponded to a high plateau of estimated CXCR4 receptor occupancy, approximately 80% (not shown). These clinical data confirmed a distinct pharmacodynamic difference between LY2510924 peptide and LY2624587 antibody in a human cancer patient population, which is consistent with the preclinical results in monkeys: LY2510924 peptide induced a robust, sustained increase in WBCs and CD34^+^ HSCs, whereas LY2624587 antibody only transiently increased WBCs and had no significant effect on CD34^+^ HSCs. As was the case with the monkey studies, these differential *in vivo* results in humans were observed despite similar, potent inhibitory effects *in vitro* and with pharmacokinetic evidence of adequate exposure to LY2624587 antibody *in vivo*.

## DISCUSSION

The SDF-1/CXCR4 axis has attracted substantial clinical interest given its key involvement in immune cell trafficking and response and tumorigenesis [1–3]. One significant finding from preclinical and clinical studies is that a variety of CXCR4 modulators, including antagonists and agonists, mobilize leukocytes and HSCs [9, 12, 18–26]. In these previous studies, the CXCR4 modulators under investigation were either a small molecule or synthetic peptide. More recently, mAbs that target CXCR4 such as LY2624587 and BMS-936564/MDX-1338 have been developed and advanced to clinical studies for cancer treatment [10, 11]. However, the potential of these CXCR4 mAbs to effectively mobilize WBCs and HSCs has been heretofore unknown. In this report, we present findings from review and assessment of both preclinical and clinical data on WBC and HSC mobilization induced by two CXCR4-targeting agents we developed, peptide antagonist LY2510924 and mAb LY2624587, in mice, monkeys, and human clinical trial patients with advanced cancer, with several important results. First, LY2510924 peptide induced a robust, sustained mobilization of WBCs including neutrophils and CD34^+^ HSCs in both preclinical species and humans, similar to small molecule inhibitor AMD3100 [18–20, 22, 24]. Second, and in contrast, LY2624587 antibody induced only transient increases in WBCs and no discernable change in CD34^+^ HSCs in monkeys, and minimal changes in humans.

Previous studies have revealed a critical role of the SDF-1/CXCR4 interaction in WBC and HSC mobilization [15, 16, 33, 34]. There are two major mechanisms involved in this function: down-regulation of cell surface CXCR4 by internalization or proteolysis, and antagonism via direct blockade of SDF-1/CXCR4 interaction. The SDF-1/CXCR4 ligand pair is involved in cell mobilization of HSC and hematopoietic precursors induced by granulocyte colony-stimulating factor or cyclophosphamide, and cell surface CXCR4 downregulation by proteolysis has been shown to be important for this function [33, 34]. Cell mobilization induced by peptide agonist CTCE-0021 has been shown to downregulate CXCR4 expression in HSCs [23]. Similarly, SDF-1 analog met-SDF-1 induced cell mobilization is associated with the ability to more profoundly downregulate surface CXCR4 than native SDF-1 [21, 35]. The small molecule inhibitor AMD3100 is characterized as a CXCR4 antagonist, and therefore is thought to induce mobilization of HSCs via receptor antagonism, though there are reports suggesting it may also alter cell surface CXCR4 expression [24, 36, 37]. CXCR4 antagonism induced leukocyte and HSC mobilization was observed with other peptide antagonists, such as BKT140 and T134 [12, 38]. Consistent with these results, we demonstrated in the present studies that LY2510924 peptide induced a dose- and time-dependent mobilization of WBCs and HSCs in mice and monkeys. Pharmacodynamic effects of LY2510924 peptide in humans have been confirmed in a clinical trial [9]. Surprisingly, LY2624587 antibody, which effectively blocked SDF-1/CXCR4 in human and monkey cells and down-regulated human cell surface CXCR4 *in vitro*, only induced a transient increase in WBCs in monkeys, and minimal increases of WBCs or no appreciable changes of HSCs in monkeys or humans. This attenuated effect of LY2624587 antibody on mobilization as compared with LY2510924 peptide and other CXCR4-targeting agents may be attributable to its biological properties or mechanism of action as a mAb.

Biochemical and cellular characterization showed that LY2510924 peptide and LY2624587 antibody, which are both SDF-1/CXCR4 antagonists, share many mechanistic similarities [8, 10]. Biochemically, both potently inhibited SDF-1 binding to human CXCR4 with similar IC_50_ values (0.08 and 0.26 nM, respectively) and inhibited monkey CXCR4 with virtually the same IC_50_ values (0.097 and 0.1 nM, respectively). Neither has shown apparent agonist activity. Cellularly, both have been shown to potently inhibit SDF-1 mediated cell migration of human histiocytic lymphoma U937 cells and cell signaling mediated by SDF-1/CXCR4 such as activation of phospho-ERK and phospho-AKT. Importantly, both compounds showed antitumor growth activity in tumor xenograft models including lymphoma and leukemia [8, 10, 27].

Despite these similarities, both agents have some distinct differences in cellular effects, as shown in the present studies and another recent report [10]. LY2624587 antibody induced cell surface CXCR4 receptor downregulation; in contrast, LY2510924 peptide was previously shown to induce no change and in the present study induced a slight increase in cell surface CXCR4 due to antagonistic function [10]. Mechanistically, it is hypothesized that LY2624587 antibody induced downregulation of cell surface CXCR4 receptor is due to the antibody facilitating receptor internalization and degradation. LY2510924 peptide is an antagonist of CXCR4 and does not induce receptor internalization; therefore, it does not induce downregulation of cell surface CXCR4 receptor. In some cases, LY2510924 peptide may induce a slight increase of the receptor due to its antagonist function, in which LY2510924 peptide binds to and locks the receptor on the cell surface. Additionally, LY2624587 antibody induced hematological tumor cell death *in vitro*, whereas LY2510924 peptide did not; the molecular mechanism underlying this difference is unknown at present.

These distinct *in vitro* properties may be related to the differential mobilization of WBCs and HSCs *in vivo*, but the mechanism is not yet understood and these differences cannot fully explain why LY2624587 antibody induced only minimal changes in WBCs and HSCs in monkeys and humans. To rule out inadequate exposure, pharmacokinetics were analyzed and showed that LY2624587 antibody achieved very high exposures in both monkeys and humans that, in fact, greatly exceeded the cellular IC_50_ of this agent. Weak immune cell mobilization by LY2624587 antibody may be a class phenomenon of mAb agents. Another CXCR4-specific mAb in clinical development, BMS-936564/MDX-1338, has similar properties including antitumor and pro-apoptotic functionality, but there are to date no published reports concerning whether this agent mobilizes WBCs or HSCs in preclinical species [11, 39]. In a phase I study in patients with chronic lymphocytic leukemia, there was no evidence of increase in the absolute number of normal lymphocytes following treatment with BMS-936564/MDX-1338 [40]. In a separate phase Ib study in patients with multiple myeloma, BMS-936564/MDX-1338 induced a median 2-fold increase in peripheral leukocytes, but the increase was transient [41]. Based on these available and limited datasets, the lack of robust mobilization of immune cells and HSCs by LY2624587 antibody (and perhaps also BMS-936564/MDX-1338) could be agent-specific or a mAb class phenomenon. A molecular mechanism explaining why LY2624587 antibody does not effectively mobilize immune cells remains under investigation in our laboratories in preclinical models.

LY2510924 peptide has been compared in preclinical studies with AMD3100, a CXCR4-targeting small molecule inhibitor that is approved by the United States Food and Drug Administration for HSC mobilization in multiple myeloma and non-Hodgkin’s lymphoma patients [25, 26]. In comparative study of preclinical models, both LY2510924 peptide and AMD3100 were shown to be CXCR4 antagonists, with LY2510924 peptide being significantly more potently antagonistic [27]. In an *in vivo* leukemia model, LY2510924 peptide showed single agent antileukemia activity [27], while an AMD3100 analog has been shown to be inactive as a single agent [42]. In clinical study, a single dose of LY2510924 peptide demonstrated sustained pharmacodynamic effects (WBC and HSC increases) up to 24 hours [9], while the effect of a single dose AMD3100 appeared to last only a few hours with the WBC count reduced to near baseline level at 24 hours [43]. These data suggest that LY2510924 peptide is a more potent CXCR4 antagonist *in vitro* and *in vivo* compared with AMD3100. Importantly, AMD3100 treatment on a chronic dosing schedule poses significant safety concerns due to compound-associated toxicity [43], and therefore may be better suited to short-term treatment in patients. This compound-associated toxicity may limit AMD3100 clinical utilization in oncology setting.

Immune checkpoint therapies, including mAbs that target CTLA-4, PD-1, or PD-L1, can enhance antitumor immune responses and have led to several recent and significant clinical advances in cancer treatment. These include durable clinical responses and long-term remissions in a subset of melanoma, lung, and other cancer patients [28, 29]. However, there are many cancer types, such as those harboring a low “mutational load,” for which a significant proportion of patients respond poorly to these therapies. Therefore, development of combination regimens to improve current cancer immunotherapies is required [29]. Since SDF-1/CXCR4 interaction is critical for retention and mobilization of immune cells, including T-cells, combination of CXCR4 inhibition with immune checkpoint inhibition improves antitumor immunity as shown in several preclinical studies. For example, in a pancreatic cancer model of combination immunotherapy, T-cells were localized to tumor microenvironment stroma and addition of CXCR4 small molecule inhibitor AMD3100 led to T-cell redistribution and rapid accumulation near tumors, enhancing the antitumor efficacy of an anti–PD-L1 mAb combination regimen [7]. In ovarian cancer models, an oncolytic vaccinia virus engineered to deliver a CXCR4 antagonist designed as a mouse Fc fragment of IgG2a inhibited tumor growth by reducing recruitment of regulatory T-cells with concomitant increased antitumor immune responses and destruction of tumor-initiating cells [31]. Treatment in these models with CXCR4 antagonists resulted in selective reduction of intra-tumor regulatory T-cells and concomitant increases in T-cell mediated antitumor immune responses.

There are currently multiple CXCR4-targeting agents in clinical development for cancer, including small molecule antagonists, peptide agonists and antagonists, and anti-CXCR4 mAbs, many of which are being evaluated in combination with immunotherapy. As we show in our studies, CXCR4-targeting peptide antagonists such as LY2510924 and CXCR4-specific mAbs such as LY2624587 have distinct functional profiles in mobilizing WBCs and HSCs in humans and preclinical species, which may be agent-specific or a class effect and is the subject of continuing investigation. This pharmacodynamic difference highlights an important potential impact on the agents’ immune effects and capacity to improve current immunotherapies. These results from two agents representing different classes of CXCR4 inhibitors, demonstrating differential effects on immune cell mobilization at therapeutic doses, may be useful to guide clinicians in the design of combination studies with immune checkpoint inhibitor or other agents for cancer immunotherapy.

## MATERIALS AND METHODS

### Experimental agents

LY2510924 is a novel cyclic peptide antagonist that blocks SDF-1 binding to CXCR4 [8, 9]. LY2624587 is a fully humanized recombinant mAb directed against CXCR4 [10]. Both agents were developed at Eli Lilly and Company (Indianapolis, IN).

### Cell lines

Human leukemia CCRF-CEM cells, B cell lymphoma Raji cells, histiocytic lymphoma U937 cells, and breast cancer MDA-MB-435 cells and mouse lymphoma 2PK-3 cells were purchased (American Type Culture Collection, Manassas, VA). Characterization of cell lines was performed (RADIL, Columbia, MO) including screening for microbial contamination or mammalian inter-species contamination. Evaluation of alleles for nine genetic markers confirmed that banked cells matched previously reported genetic profiles. Cells were grown in RPMI 1640 or DMEM supplemented with 10% (v/v) FBS, passaged twice weekly for <2 months, and maintained in an incubator at 37°C with 5% CO_2_.

### SDF-1/CXCR4 binding assay

The SDF-1/CXCR4 binding assay used human leukemia CCRF-CEM cells that express endogenous CXCR4 and [125I]-labeled recombinant human SDF-1α (PeproTech EC Ltd., London, UK) as previously described [8]. The same assay was conducted for breast cancer MDA-MB-435 cells transfected with monkey CXCR4 and mouse lymphoma 2PK-3 cells that express endogenous mouse CXCR4. Plates were counted in a 1450 Microbeta Liquid Scintillation and Luminescence Counter (Wallac) in SPA mode.

### CXCR4 expression and cell death analysis with flow cytometry

Methodology for cell surface CXCR4 downregulation via internalization was previously reported for LY2624587 antibody [10]. In the present study, effects of both LY2510924 peptide and LY2624587 antibody on CXCR4 expression were evaluated using a phycoerythrin (PE)-conjugated CXCR4 mAb (R&D Systems) that does not compete with either agent for CXCR4 binding. Briefly, 1x10^5^ U937 or Raji cells were seeded in glass-bottom dishes (MatTek) and cultured overnight. Cells were treated with 1 μM LY2510924 peptide or 4 μg/mL LY2624587 antibody for 2 hours at 37°C, fixed with 2% formaldehyde for 10 minutes, incubated with PE-CXCR4 mAb or isotype control for 30 minutes, then analyzed for CXCR4 expression and fluorescence intensity via LSR II flow cytometry (BD Biosciences, San Diego, CA). Anti-CXCR4 mAbs were purchased from Santa Cruz Biotechnology (Dallas, TX).

LY2510924 and LY2624587 effects on induction of cell death were evaluated in Raji cells cultured with or without 10 μg/mL LY2624587 antibody or 2.5 μM LY2510924 peptide for 48 hours. Briefly, cells were washed and seeded in a 96-well plate at 3x10^6^ cells/150 μL complete media per well; the assay was conducted at 4°C until flow cytometry analysis. Cells were Fc-blocked; washed; incubated in complete media with AmCyan live/dead dye (1:500 dilution), anti–CXCR4-FITC (1:5 dilution), or control isotype-FITC for 45 minutes; then washed in complete media and resuspended in FACS buffer (PBS plus 2% FBS) for labeling with cell population-specific mAbs. Analysis was done with LSR II flow cytometry (BD) that was pre-optimized under compensation with AmCyan and FITC. Fluorescence intensity from LSR II images was analyzed with FlowJo software; AmCyan live/dead dye stained cells (dead cells) were sorted with FlowJo software from LSR II images and calculated as percentage of total cells analyzed.

### Mouse model of HSC and WBC mobilization

Pathogen-free, 5- to 6-week-old female C57BL/6 mice (Taconic) were housed in the Eli Lilly and Company animal facility for ≥1 week prior to any experiment to allow stabilization of peripheral blood cell counts. Mouse HSC and WBC mobilization were evaluated as previously described [38]. Briefly, at multiple time points following a single subcutaneous injection with LY2510924 peptide (0.06, 0.185, 0.56, 1.67, or 5 mg/kg) or its acetate salt form LSN2534820 (0.03, 0.1, 0.3, 1, or 3 mg/kg), mice were sacrificed by CO_2_ asphyxiation and cervical dislocation and peripheral blood was collected via cardiac puncture with EDTA-coated syringes and tubes. Complete blood cell analysis was performed on a Hemavet Mascot hematology analyzer (Drew Scientific Group, Dallas, TX). Colony-forming units (CFU) were used to count peripheral HSCs. Briefly, 100 μL of whole blood was lysed with 1.5 mL red blood cell lysing buffer (BD). After washing with PBS, remaining blood cells were collected by centrifugation and mixed with 2 mL CFU growth medium as previously described [38]. The mixture was transferred to a 35-mm dish and cultured at 37°C with 5% CO_2_ for 10 to 14 days. Colonies were counted under a Nikon inverted microscope.

### Monkey model of HSC and WBC mobilization

Male and female Cynomolgus monkeys (Charles River) age 2 to 4 years and weight 2.5 to 4 kg were subcutaneously dosed with LY2510924 peptide or its salt acetate form LSN2534820 (0.01, 0.1, or 1 mg/kg daily for 4 consecutive days or 10 mg/kg single injection) or CXCR4 mAb LY2624587 (10 or 30 mg/kg single injection). Whole blood samples were collected from each animal pre-dose for baseline counts, and post-dose at multiple time points up to 24 hours post first dose (day 1) and last dose (day 4) for cell counts. WBCs and neutrophils were analyzed via routine blood analysis in a clinical pathology laboratory. Counts of CD34^+^ cells in peripheral blood were enumerated using flow cytometry per a protocol developed in our laboratory. Briefly, 20 μL of a stock staining solution (1/3 anti-CD45-perCP, 1/3 anti-CD34-PE, and 1/3 Syto16 to 1 μM final) was added to Procount tubes with CaliBRITE beads (BD) for cell quantitation. Tubes containing stock staining solution were diluted with 25 μL whole blood plus 25 μL RPMI 1640 + 0.1% BSA, incubated for 15 minutes at room temperature in the dark followed by addition of 450 μL 1x BD FACS lysing solution, incubation for 30 minutes, then flow cytometry analysis with a Beckman Coulter FC500. Gating/region strategies were defined by SS versus nuclear dye (lymphocytes) and SS versus CD45 (CD45-negative/dim cells), and combined regions were viewed in a dot plot of CD34 versus nuclear dye (counting both CD34-negative/dim and CD34^+^ cells in the final plot). Beads were defined in the SS versus perCP plot to count CD34^+^ cells/μL whole blood as follows:

[# CD34^+^ cells / # beads] x [beads per test / test volume] x dilution factor.

### Human clinical studies of HSC and WBC mobilization

The phase I clinical trial of LY2510924 peptide in 45 patients (N = 39 in pharmacodynamic dataset) with advanced cancer was previously reported [9]. The phase I clinical trial of LY2624587 antibody in 56 patients (N = 23 in pharmacodynamic dataset) with advanced cancer was conducted as reported (NCT01139788). Both were multicenter, nonrandomized, dose escalation studies with 28-day treatment cycles. LY2510924 peptide was administered daily by subcutaneous injection up to the maximum dose of 30 mg/day. LY2624587 antibody was administered as weekly intravenous infusions up to the maximum dose of 900 mg once-weekly. Blood samples for cell counts were obtained at baseline, pre-dose, and post-dose time points. LY2510924 peptide is currently in phase II clinical studies, and LY2624587 antibody is on hold for further clinical development after completion of phase I clinical study.

### Systemic exposure analysis of CXCR4 mAb LY2624587

Serum LY2624587 antibody concentrations in Cynomolgus monkeys and human clinical trial patients were determined using a selective and validated ELISA per a protocol developed at Eli Lilly and Company.

### Statistical analyses

Mean values are shown for data presentations, with error bars representing standard error of the means. Except where indicated otherwise, data were analyzed and plotted with JMP software (SAS Institute), including one-way ANOVA for *in vivo* mouse WBC and neutrophil count changes. Treatment effect was evaluated with the Student *t*-test; a treatment difference was statistically significant if *P* ≤ 0.05. Due to the exploratory nature of these analyses, no adjustments for multiple comparisons were done.

### Ethics approvals

Animal study protocols were approved by the Animal Care and Use Committee of Eli Lilly and Company. Human clinical trials were conducted in accordance with good clinical practice and ethical standards of the Declaration of Helsinki and were approved by Institutional Review Boards; written informed consent was obtained from each patient before enrollment.

## SUPPLEMENTARY MATERIALS FIGURE



## Abbreviations

ANOVA: analysis of variation
BSA: bovine serum albumin
CFU: colony-forming units
CO_2_: carbon dioxide
CXCR4: C-X-C receptor 4
DMEM: Dulbecco’s Modified Eagle’s Medium
ED_50_: median effective dose
EDTA: ethylenediaminetetraacetic acid
ELISA: enzyme-linked immunosorbent assay
FBS: fetal bovine serum
FACS: fluorescence activated cell sorting
FITC: fluorescein isothiocyanate
HSC: hematopoietic stem cell
IC_50_: half-maximal inhibitory concentration
Ki: inhibitor constant
mAb: monoclonal antibody
NS: not significant
PBS: phosphate-buffered saline
PE: phycoerythrin
QDx1: single dose
QDx4: once daily for 4 days
RPMI: Roswell Park Memorial Institute (culture medium)
SDF-1: stromal cell-derived factor-1
v/v: volume/volume (percent)
WBC: white blood cell
