# Supplementary material for: Distinct mobilization of leukocytes and hematopoietic stem cells by CXCR4 peptide antagonist LY2510924 and monoclonal antibody LY2624587
Source: Oncotarget. 2017 Oct 10;8(55):94619–34. doi: 10.18632/oncotarget.21816 (PMC5706900; doi:10.18632/oncotarget.21816)
Supplement: Supplementary file 1 [file oncotarget-08-94619-s001.pdf]

Distinct mobilization of leukocytes and hematopoietic stem cells by CXCR4 peptide antagonist LY2510924 and monoclonal antibody LY2624587

SUPPLEMENTARY MATERIALS

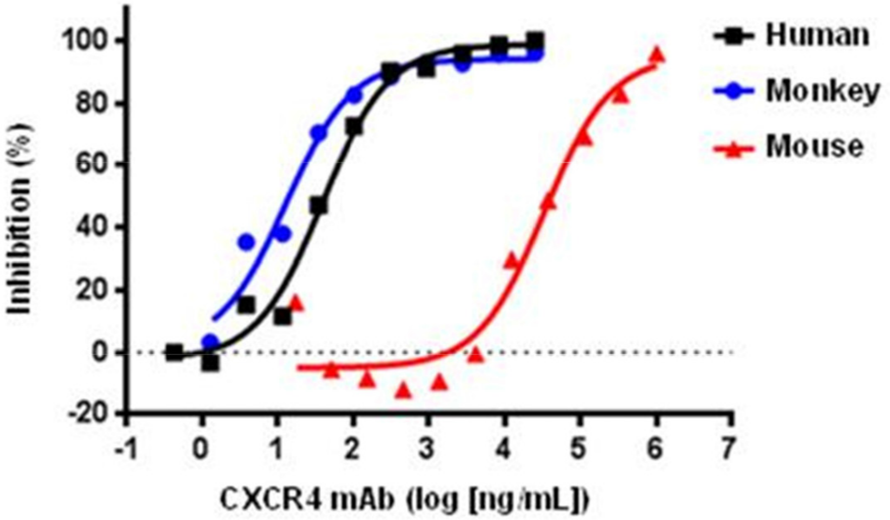

Supplementary Figure 1: LY2624587 antibody inhibition of SDF-1/CXCR4 binding. LY2624587 (CXCR4 mAb) mediated inhibition of binding in human, monkey, and mouse cells. Human cells = leukemia CCRF-CEM cells with high-level expression of human CXCR4, monkey cells = MDA-MB-435 cells stably transfected with monkey CXCR4, and mouse cells = 2PK-3 lymphoma cells with high-level expression of mouse CXCR4.
